# Food-dependent exercise-induced anaphylaxis to mango

**DOI:** 10.1016/j.jacig.2026.100725

**Published:** 2026-04-30

**Authors:** Simon Doré, Louis Marois

**Affiliations:** a Faculty of Medicine, Laval University, Québec City, Québec, Canada; b Immunology and Allergy Service, CHU de Québec, Québec City, Québec, Canada; c CHU de Québec Research Center, Laval University, Québec City, Québec, Canada

**Keywords:** Food dependent exercise-induced anaphylaxis, anaphylaxis, food allergy, mango allergy, fruit allergy

## Abstract

This rare case of mango-dependent exercise-induced anaphylaxis underscores the role of cofactors and illustrates the diagnostic challenges in identifying uncommon food-dependent exercise-induced anaphylaxis triggers.

Food-dependent exercise-induced anaphylaxis (FDEIA) is a rare condition in which symptoms of anaphylaxis occur only when ingestion of a culprit food is followed by physical activity.^1^ Individually, exercise and food are tolerated, but their combination within 4 hours can trigger anaphylaxis. Symptoms usually appear within 1 hour (98%) but can be delayed up to 4 (rarely 6) hours. Manifestations include urticaria and/or angioedema plus respiratory (dyspnea, wheezing, stridor, hypoxemia) and/or cardiovascular compromise (hypotension, collapse, syncope). About 16.6% of patients present with isolated urticaria/angioedema without systemic involvement, broadening the spectrum to “food-dependent exercise-induced allergic reactions.”^1^

Wheat is the most common culprit food, followed by vegetables, seafood, and legumes. Fruits are uncommon triggers (<6% of cases), with peach, cherry, nectarine, and mango reported in very rare cases.^1^^,^^2^ Common cofactors include nonsteroidal anti-inflammatory drugs (NSAIDs), menstruation, and alcohol.^1^ Here, we report a case of mango (*Mangifera indica*)-dependent FDEIA; review its features; and discuss pathophysiology, diagnostic pitfalls, and management.

## Case report

A 21-year-old woman experienced 2 food-dependent exercise-induced allergic reactions after mango ingestion, of which only the second fulfilled the diagnostic criteria for anaphylaxis. The patient’s history included atopic dermatitis, allergic rhinitis, mild asthma, and possible pineapple allergy. Aeroallergen sensitization was documented by skin prick testing, the results of which were positive to house dust mite, cat, dog, grass pollen mix, tree pollen mix, and dandelion. At age 18 years, shortly after eating mango and exercising, she developed urticaria, pruritus, and subjective weakness without respiratory, gastrointestinal, or cardiovascular involvement; her symptoms resolved with oral diphenhydramine.

The patient’s second reaction occurred 2 years later; 1 hour after eating fresh mango, she went jogging. After 35 to 40 minutes, she developed pruritus, dyspnea, nausea, vomiting, and presyncope. She collapsed in the park but managed to call emergency services; on arrival, the paramedics noted sinus tachycardia without measurable blood pressure. Intramuscularly injected epinephrine (0.5 mg) resolved the patient’s symptoms. In the emergency department, her vitals stabilized (O_2_ saturation, 97%; heart rate, 88 beats per minute; respiration rate, 17 breaths per minute; and blood pressure, 105/72 mm Hg); her residual lip edema remained, but her rash had resolved. That morning, she had taken naproxen for menstrual pain. She had tolerated mango ingestion without exercise and exercise without food between episodes.

During allergy consultation 2 months later, the results of skin prick testing were positive to fresh mango pulp (5 mm; histamine 3 mm) and negative to pineapple and canned mango (3 mm). Serum-specific IgE was undetectable (<0.35 kUA/L) for mango, pineapple, wheat, and omega-5-gliadin. The patient’s total IgE level was 47.5 kU/L, and her baseline serum tryptase level was normal (5.3 μg/L). Spirometry showed mild asthma with a positive methacholine challenge result (provocative concentration causing a 20% drop in FEV_1_ value from baseline, 5.84 mg/mL). Oral mango challenge using approximately 100 g of fresh mango pulp (∼125 mL) was administered in incremental doses every 15 minutes over 60 minutes under medical supervision, followed by a 2-hour observation period; the result was negative in the absence of exercise.

The patient was advised to avoid mango ingestion within 6 hours before and 4 hours after exercise, avoid NSAIDs near physical activity, and carry an epinephrine autoinjector. During 18 months of follow-up, she remained symptom-free with no recurrence of food-dependent reactions or anaphylaxis.

## Discussion

This is the second reported case of mango-dependent FDEIA.^2^ In this case, the following cofactors were decisive: mango combined with exercise caused mild symptoms, whereas additional cofactors (NSAID and menstruation) precipitated anaphylaxis.

Differential diagnoses were considered. Tolerance to exercise alone and mango ingestion without exercise argued against cholinergic urticaria and isolated food allergy. Mast cell disorders were unlikely given the patient’s normal baseline serum tryptase level, absence of recurrent spontaneous symptoms, and lack of other clinical features suggestive of clonal mast cell disease or hereditary alpha-tryptasemia.

During exercise, the proposed mechanisms for FDEIA include increased gastrointestinal permeability and reduced gastric acid secretion, facilitating allergens absorption and mast cell activation^1^^,^^3^; NSAIDs may further lower activation thresholds.^1^^,^^4^ Measurement of serum tryptase level can support the diagnosis of anaphylaxis when it is elevated or identify underlying mast cell disorders, but normal levels do not exclude FDEIA, as illustrated in this case.

The patient’s positive skin test results but negative serum IgE result are consistent with the poor sensitivity of current mango ImmunoCAP assays.^5^ The absence of a formal food exercise challenge represents a methodologic limitation, but such challenges have limited sensitivity and carry inherent risk^1^; the diagnosis was therefore based on a consistent, reproducible clinical history with documented cofactor dependence. Given the patient’s aeroallergen sensitization and the fruit trigger, pollen-food cross-reactivity mediated by labile panallergens such as profilin was considered. However, the clinical phenotype, negative result of testing for serum-specific IgE, and positive result of prick-by-prick testing to fresh mango suggest sensitization to heat-labile or poorly characterized mango components rather than to lipid transfer proteins.

Rare fruit triggers have also been reported in isolated cases. Rosaceae fruits such as peach and cherry have caused anaphylaxis in adolescents despite a negative result of testing for IgE to lipid transfer proteins, thus suggesting other allergenic components.^6^ Nectarine, a peach variant, has similarly been reported as a trigger with weak skin test positivity but negative component-resolved diagnostics.^7^ These observations highlight the fact that FDEIA can occasionally involve uncommon fruits, with mechanisms that may differ from the well-characterized wheat- or shellfish-related forms.

Management relies on avoidance of trigger-cofactor combinations. Patient education, including training in recognition of early anaphylaxis symptoms and correct use of epinephrine autoinjectors, is a cornerstone of care (Table I).^1^^,^^3^

This case of mango-dependent FDEIA underscores the critical role of cofactors, the diagnostic limitations of current IgE assays, and the need for awareness of rare fruit allergens. Reporting such cases expands the clinical spectrum of FDEIA and reinforces the importance of patient education, strict avoidance, and emergency preparedness.

Statement on consent: Written informed consent was obtained from the patient for publication of this case report and any accompanying clinical information.

**Table I:** Practical clinical recommendations for patients with FDEIA

Clinical recommendations for patients with FDEIA
Avoidance of culprit food
4-6 h before exercise
1-4 h after exercise
Avoidance of augmenting factors (acetylsalicylic acid/NSAIDs, alcohol) surrounding exercise
Access to self-injectable epinephrine when exercising
Patient education and an anaphylaxis action plan

## Disclosure statement

Disclosure of potential conflict of interest: The authors declare that they have no relevant conflicts of interest.

## References

[1] Kulthanan K., Ungprasert P., Jirapongsananuruk O., Rujitharanawong C., Munprom K., Trakanwittayarak S. (2022). Food-dependent exercise-induced wheals, angioedema, and anaphylaxis: a systematic review. J Allergy Clin Immunol Pract.

[2] Toya T., Kagami K., Adachi T. (2019). Friend or foe: food-dependent exercise-induced anaphylaxis associated with acute coronary syndrome aggravated by adrenaline and aspirin: a case report. Eur Heart J Case Rep.

[3] Srisuwatchari W., Kanchanaphoomi K., Nawiboonwong J., Thongngarm T., Sompornrattanaphan M. (2023). Food-dependent exercise-induced anaphylaxis: a distinct form of food allergy-an updated review of diagnostic approaches and treatments. Foods.

[4] Mohamed S., Thalappil S., Mohamed Ali R. (2024). A case report of food-dependent exercise-induced anaphylaxis (FDEIA) treated with omalizumab. Front Allergy.

[5] Zhao L., Xie H., Wang X., Liu M., Ma T., Fu W. (2023). Molecular characterization of allergens and component-resolved diagnosis of IgE-mediated mango fruit allergy. Allergy.

[6] Bianchi A., Di Rienzo Businco A., Bondanini F., Mistrello G., Carlucci A., Tripodi S. (2011). Rosaceae-associated exercise-induced anaphylaxis with positive SPT and negative IgE reactivity to Pru p 3. Eur Ann Allergy Clin Immunol.

[7] Miceli Sopo S., Monaco S., Giorgio V., Calvani M., Mistrello G., Onesimo R. (2013). Food-dependent exercise-induced anaphylaxis (FDEIA) by nectarine in a paediatric patient with weakly positive nectarine prick-by-prick and negative specific IgE to Pru p 3. Allergol Immunopathol (Madr).

